# Mortality Risk Factors for Coronavirus Infection in Hospitalized Adults in Brazil: A Retrospective Cohort Study

**DOI:** 10.3390/ijerph192114074

**Published:** 2022-10-28

**Authors:** Rafael Alves Guimarães, Raquel Silva Pinheiro, Hellen da Silva Cintra de Paula, Lyriane Apolinário de Araújo, Ingrid Aline de Jesus Gonçalves, Charlise Fortunato Pedroso, Adriana Oliveira Guilarde, Geraldo Andrade de Oliveira, Karla de Aleluia Batista

**Affiliations:** 1 Faculty of Nursing, Federal University of Goiás, Goiânia 74605-080, GO, Brazil; 2 Federal Institute of Education, Science and Technology of Goiás, Campus Goiânia Oeste, Goiânia 74395-160, GO, Brazil; 3 Institute of Tropical Pathology and Public Health, Federal University of Goiás, Goiânia 74605-050, GO, Brazil; 4 Federal Institute of Education, Science and Technology of Goiás, Valparaíso Campus, Valparaíso 72876-601, GO, Brazil

**Keywords:** COVID-19, mortality, risk factors

## Abstract

Background: The COVID-19 pandemic has presented high morbidity and mortality, with associated high socioeconomic costs. Brazil ranks third in the number of COVID-19 cases, behind only India and the United States. Objective: To analyze risk factors for mortality in adults hospitalized with COVID-19 in Brazil. Methods: Observational retrospective cohort study including data from all Brazilian states and regions. The study included information from 468,226 in-hospital patients from all regions of Brazil from 1 January 2021 to 31 July 2021. Data from the influenza epidemiological surveillance system were used. The participants were adults hospitalized with COVID-19. A Cox regression model was used to analyze factors associated with mortality in adults with COVID-19. Results: The in-hospital mortality lethality was 37.5%. The risk factors associated with COVID-19 mortality were older age, with a linear increase with increments in age, male sex, black or mixed race, low education level, comorbidities, use of ventilatory support, and living in the southeast, north, or northeast regions of the country. Conclusions: Our results illustrate the severity of the COVID-19 pandemic in Brazil and reinforce that policies and practices to deal with this disease should focus on groups and regions with higher risk, whereas public policies should promote nonpharmacological measures and vaccination in the Brazilian population.

## 1. Background

The COVID-19 pandemic, caused by severe acute respiratory syndrome coronavirus 2 (SARS-CoV-2), has led to high morbidity and mortality, and it had high socioeconomic costs [1,2]. As of 4 October 2022, the World Health Organization (WHO) estimated a total of 615,777,700 million confirmed cases and 6,527,129 million deaths caused by COVID-19 worldwide [3]. The severity of the pandemic situation led the countries to develop different preventive actions to reduce the spread of the virus, including social distancing and entire lockdown [4].

Brazil ranks third in the number of COVID-19 cases, behind only India and the United States [3]. The first case of COVID-19 in the country was reported on 25 February 2020, and data from the Ministry of Health show that over 34 million people have been infected with SARS-CoV-2, and over 686,000 deaths have occurred as a result [5]. These values may be underestimated due to the low levels of testing in Brazil and the underreporting of cases and deaths, aggravated by the emergence of new variants of SARS-CoV-2, a predominance was observed of B.1.1.33 and B.1.1.28 variants from the pandemic beginning in September 2020, followed by zeta from October 2020 to February 2021, gamma from mid-February 2021 to September 2021, delta from October 2021 to December 2021, and omicron variants from January 2022 until now [6].

Additionally, the political crisis that hindered the implementation of early strategies to increase vaccination coverage and effective nonpharmacological interventions at the federal level, and the nonadherence to preventive measures and vaccine hesitancy among the population may also have contributed to reducing the amount of information about contamination and COVID-19 related deaths [7,8,9].

Studies have shown that multiple variables are associated with increased risk of developing fatal COVID-19. There is evidence that male, less educated, black or mixed-race individuals are more likely to die [1,10,11,12,13,14]. Studies also show that the risk of mortality increases linearly with increasing age, impacting the elderly more [1,10,11,12,13,14]. This is also true for individuals with comorbidities, such as cardiovascular disease, diabetes mellitus, chronic liver disease, chronic kidney disease, chronic neurological disease, and obesity, regardless of age [10,15,16,17]. Unvaccinated people and those with incomplete vaccination status have a greater severity of the disease and, consequently, longer hospital stays and a higher mortality rate [18].

In Brazil, fatality rates are higher in less socioeconomically developed regions, such as in the north and northeast, due to the lower access to health services and hospital and intensive care unit (ICU) beds and a limited number of health care professionals [1,19].

Despite the increase in research, few studies investigated the effects of some comorbidities, such as chronic liver disease and neurological disease, on mortality in hospitalized patients after adjusting for other covariates. Moreover, little evidence is available concerning each COVID-19 sign and symptom as a mortality predictor. Finally, the roles of race and education on mortality among hospitalized patients have been poorly investigated, possibly due to the low quality of these variables in the databases. However, the analysis of these factors can contribute to the development of effective public policies focused on subgroups of higher vulnerability for fatal COVID-19 and cope with the current and future pandemics. Furthermore, understanding these factors can support the clinical management of individuals, especially in terms of interventions to increase the survival of patients with COVID-19. Thus, this study aimed to analyze risk factors for mortality in adults hospitalized with COVID-19 in Brazil. We assessed the effects of sociodemographic variables, comorbidities, and the presence of signs and symptoms of COVID-19 on overall mortality.

## 2. Material and Methods

### 2.1. Design

We conducted an observational retrospective cohort study that analyzed the risk factors for mortality in adults hospitalized with COVID-19 in Brazil.

### 2.2. Context

**Figure 1:**
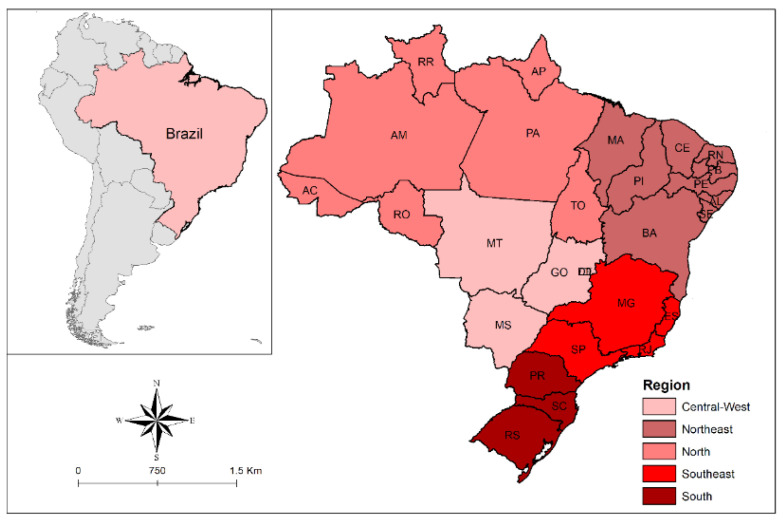
Study location (Brazil, regions, and states).

The study included data from all Brazilian states and regions. Brazil is the largest country in Latin America in size and population, covering 8,510,345.538 km^2^ and with an estimated population of 213.3 million in 2021 [20]. The country has a Human Development Index of 0.765, occupying the 84th position in the world ranking, a life expectancy at birth of 75.9 years and expected years of schooling of 15.4 years [21]. The country has 5570 cities that are distributed in 27 federation units (states), which, in turn, are grouped into 5 major regions (Figure 1).

### 2.3. Data Source

We used data extracted from the Influenza Epidemiological Surveillance Information System (SIVEP-Gripe) of the Brazilian Ministry of Health. This is a health information system that contains information about cases and deaths of individuals hospitalized due to severe acute respiratory syndrome (SARS). Every case of SARS is individually and continuously reported in the system by health professionals. The SIVEP-Gripe is part of the surveillance network for influenza and other respiratory viruses, including SARS-CoV-2 [5].

The cases of SARS reported follow the definition of the Brazilian Ministry of Health. In adults, a SARS case is defined by the presence of signs or symptoms consistent with flu-like syndrome (FLS), accompanied by dyspnea or respiratory distress or persistent chest pressure or cyanosis or oxygen saturation <95% on room air. In turn, FLS is defined as an acute respiratory condition characterized by at least two of the following signs and symptoms: fever (measured or reported), chills, sore throat, headache, cough, runny nose, smell disturbances, or taste disturbances [5].

In a nutshell, SIVEP-Gripe records hospitalized cases with SARS in Brazil’s public health system (Sistema Único de Saúde-SUS) and in private health care facilities. The information is collected by health professionals in the standardized notification form, which includes data on sociodemographic characteristics, signs and symptoms of respiratory infections, comorbidities, and evolution (cure, death from the diagnosed infection, or death from another cause), among other variables of interest (e.g., presence of anosmia and ageusia). In the surveillance network, respiratory infections such as SARS-CoV-2 are confirmed in the laboratory using serological tests such as enzyme-linked immunosorbent assay, viral isolation, or the detection of viral nucleic acid RNA by real-time polymerase chain reaction (RT-PCR) [5].

Data quality (e.g., identification of duplicates, completeness, and consistency) is checked at the local level automatically, and the data are transferred to the state and federal levels. This study only included data from the population of interest included the database between 1 January and 31 July 2021.

### 2.4. Population

The study population was composed of individuals aged 18 years or older, of both sexes, who had a positive laboratory diagnosis for SARS-CoV-2 infection and were hospitalized between 1 January and 31 July 2021. In the present study, individuals with COVID-19 were those with a positive qualitative diagnosis using the RT-PCR test, which are processed in public health, hospital, and private laboratories in Brazil.

### 2.5. Variables

#### 2.5.1. Outcome

Outcome was the time until in-hospital death from all causes, defined as the time between the date of hospital admission (time of onset) and the failure variable. This variable was calculated in full days. Failure was categorized as 0 for alive or 1 for dead. Individuals in the cohort were censored when (i) they were discharged from the hospital before the end of follow-up and (ii) survived at the end of 90 days of hospital stay.

#### 2.5.2. Exposure Variables

The extracted sociodemographic, clinical, and health assistance data were used as exposure variables:

(i) Sociodemographic variables: age group, categorized as 18–29 years, 30–39 years, 40–49 years, 50–59 years, 60–69 years, or 70 years or old; sex (male or female); (iii) race classification (white, mixed race, black, or others, which included the Asian or indigenous categories due to the small number of observations) according to the Brazilian Institute of Geography and Statistics (Instituto Brasileiro de Geografia e Estatística) [22]; level of education, categorized into five levels (illiterate, elementary school [ES-1], elementary school [ES-2], high school or higher education) [19], and region of the country (south, southeast, central-west, northeast, or north).

(ii) Comorbidities: Categorized as no or yes. The following comorbidities were considered: heart disease, diabetes mellitus, obesity, chronic kidney disease, chronic neurological disease, pneumopathy, asthma, immunodepression, chronic liver disease, and chronic hematological disease, and other comorbidities. The variable multiple comorbidities were also analyzed, defined as the presence of two or more concurrent comorbidities [23]. The largest proportion of these variables was obtained by patients’ self-report.

(iii) Health assistance: ICU admission and ventilation requirement (no; yes, not invasive; or yes, invasive). The main signs or symptoms predictive of mortality in hospitalized patients were analyzed. Each sign or symptom was categorized as no or yes: fever, cough, sore throat, dyspnea, respiratory distress, oxygen saturation <95%, diarrhea, vomiting, abdominal pain, fatigue, alterations in smell, and loss of taste.

### 2.6. Statistical Analysis

The data were analyzed in the R Studio software. The Anderson–Darling test was performed to analyze the normality of the quantitative variables. Descriptive summary statistics were performed: qualitative variables were presented as absolute (n) and relative (%) frequency and quantitative variables as median and interquartile range (IQR) due to the absence of normality. We also estimated the case fatality rate, calculated by the number of individuals hospitalized with the outcome (all-cause death from COVID-19) and the number of positive cases on RT-PCR multiplied by 100. The case fatality rate was estimated by all the explanatory variables analyzed.

We used the Cox proportional hazards model to analyze the risk factors for mortality in adults hospitalized with COVID-19. Initially, bivariate analysis was performed between the outcome variable and explanatory variables. The groups had their survival functions compared by log-rank test, and the survival function was graphically represented by Kaplan–Meier curves. This analysis was complemented by bivariate Cox regression analysis, obtaining the crude hazard ratio (cHR) and 95% CI. Variables with a *p*-value < 0.20 in the bivariate analysis were included in the Cox proportional hazards model to adjust for potential confounding variables. Cox proportionality analysis was performed for the final model. The magnitude of the effect of the final model was obtained by the adjusted hazard ratio (aHR), regression coefficient (β), and respective 95% CI. The significance level was obtained with the Wald test. Kaplan–Meier survival curves and log-rank tests were used to compare the survival functions for each sign or symptom of COVID-19 at baseline, and then Poisson regression analysis was performed to analyze the signs and symptoms predictive of mortality; *p*-values < 0.05 were considered statistically significant.

### 2.7. Ethical Aspects

This study used data available in secondary and public databases, without identifying participants and other sensitive variables. Therefore, this study was not submitted to the Research Ethics Committee, according to Resolution number 510/2016 of the National Health Council of the Ministry of Health.

### 2.8. Patient and Public Involvement

The patients had no role in the design, recruitment, or conduct of the study.

### 2.9. Data Availability

The data used in this study are publicly accessible and were extracted from the following website: https://opendatasus.saude.gov.br/dataset/bd-srag-2021 accessed on 16 August 2021.

## 3. Results

### 3.1. Population Characteristics

In this study, 468,226 hospitalizations of adults with laboratory-confirmed COVID-19 were included. The median age of hospitalized patients was 53 years (IQR = 41−75); most were men (55.8%) and white (57.2%); almost half (48.8%) had low level of education (illiterate, 4.6%; elementary school 1, 24.7%; elementary school 2, 19.5%). Most lived in southeast Brazil (54.5%) (Table 1).

The most frequent signs and symptoms of COVID-19 were oxygen saturation <95% (81.3%), dyspnea (83.3%), cough (79.0%), respiratory distress (70.6%), and fever (66.5%). The most prevalent comorbidities were heart disease (65.1%), diabetes mellitus (48.3%), and obesity (27.1%). The prevalence of multiple comorbidities was 51.2%. A total of 40.4% of patients were admitted to ICU, and 24.8% required invasive ventilatory support (Table 2).

### 3.2. Risk Factors for COVID-19 Mortality

The median follow-up time for the cohort was 57 days (IQR = 45–69; range = 0–90), and the overall in-hospital case fatality rate was 37.5% (175,684/292,500 patients). The median survival time of the cohort was 19.0 days (95% CI: 18.9–19.1).

Table S1 shows the population characteristics disaggregated by survivor subgroups and death. Table S2 shows the median survival time and 95% CI per subgroup, in addition to the survival curve comparison test (log-rank).

**Figure 2:**
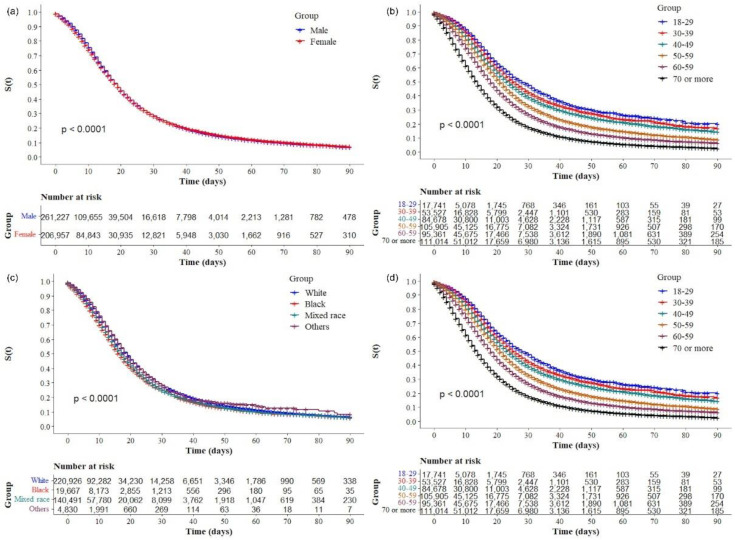
Kaplan–Meier survival curves for hospitalized COVID-19 adult patients in Brazil stratified by (**a**) sex at birth; (**b**) age group; (**c**) race; and (**d**) education.

**Figure 3:**
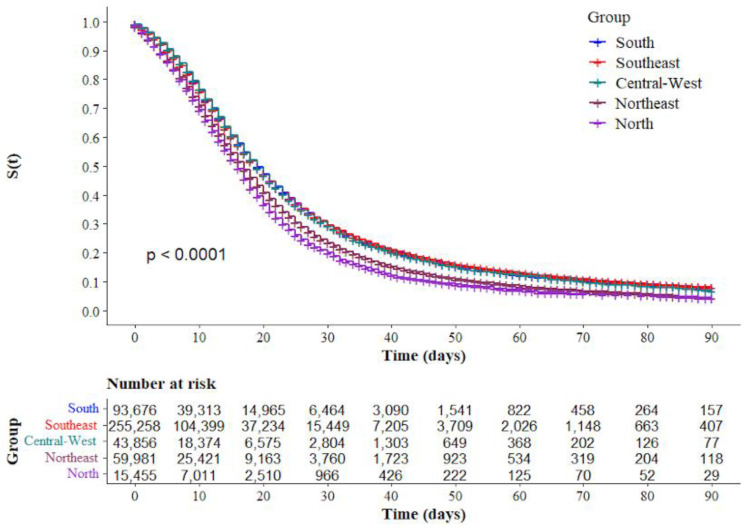
Kaplan–Meier survival curves for hospitalized COVID-19 adult patients in Brazil stratified by region.

**Figure 4:**
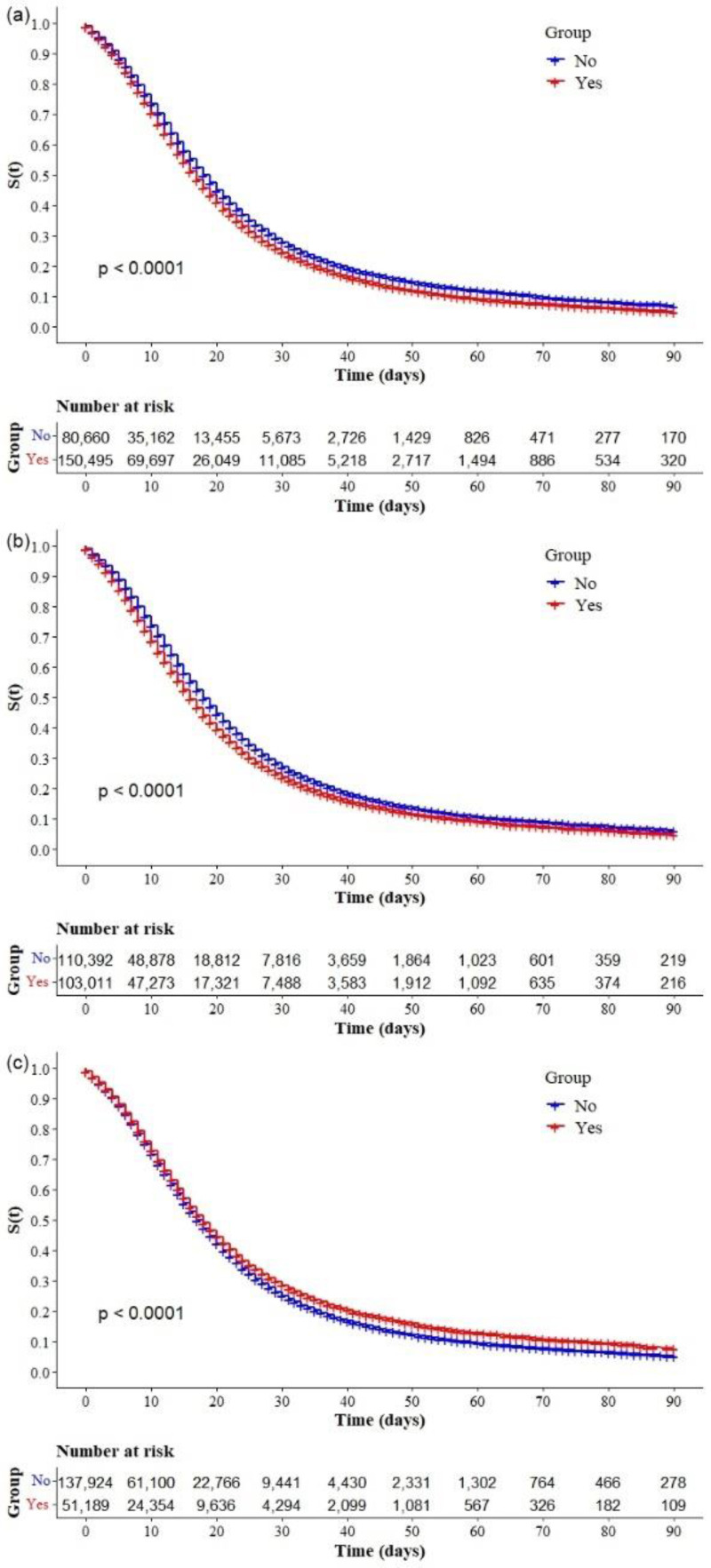
Kaplan–Meier survival curves for hospitalized COVID-19 adult patients in Brazil. Among all hospitalizations, the survival rate was stratified by the following comorbidities: (**a**) cardiopathy; (**b**) diabetes; and (**c**) obesity.

**Figure 5:**
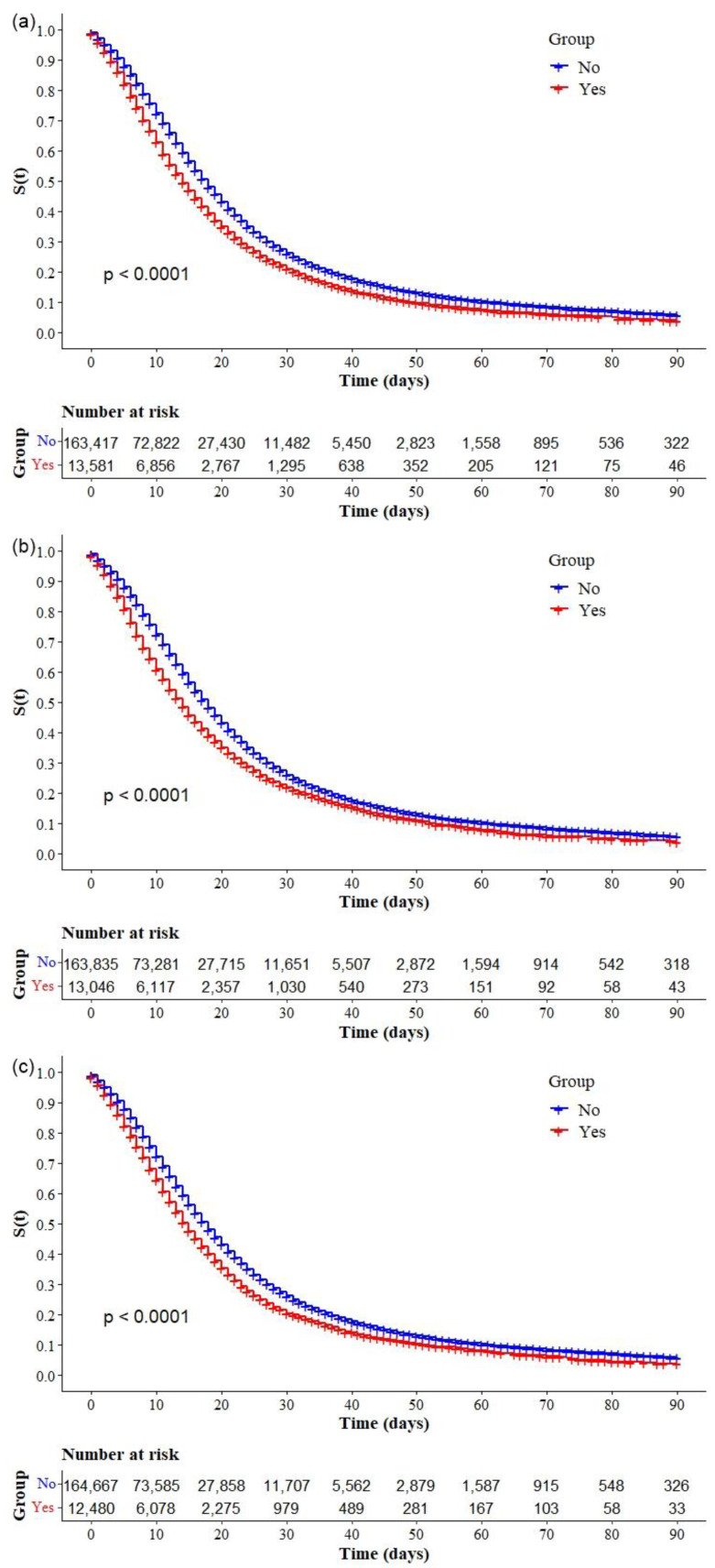
Kaplan–Meier survival curves for hospitalized COVID-19 adult patients in Brazil. Among all hospitalizations, the survival rate was stratified by the following comorbidities: (**a**) chronic kidney disease; (**b**) chronic neurological disease; and (**c**) pneumopathy

**Figure 6:**
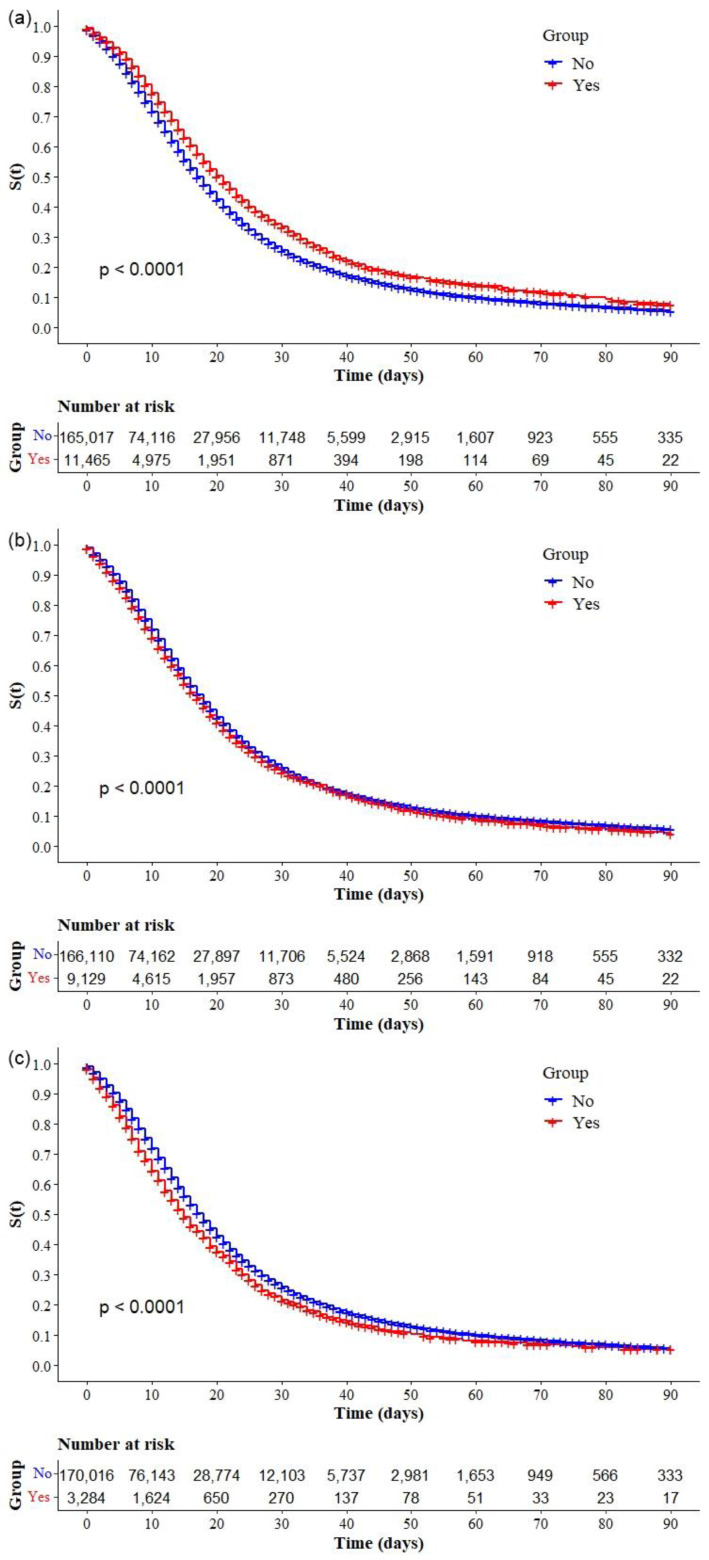
Kaplan–Meier survival curves for hospitalized COVID-19 adult patients in Brazil. Among all hospitalizations, the survival rate was stratified by the following comorbidities: (**a**) asthma; (**b**) immunodepression; and (**c**) chronic liver disease.

**Figure 7:**
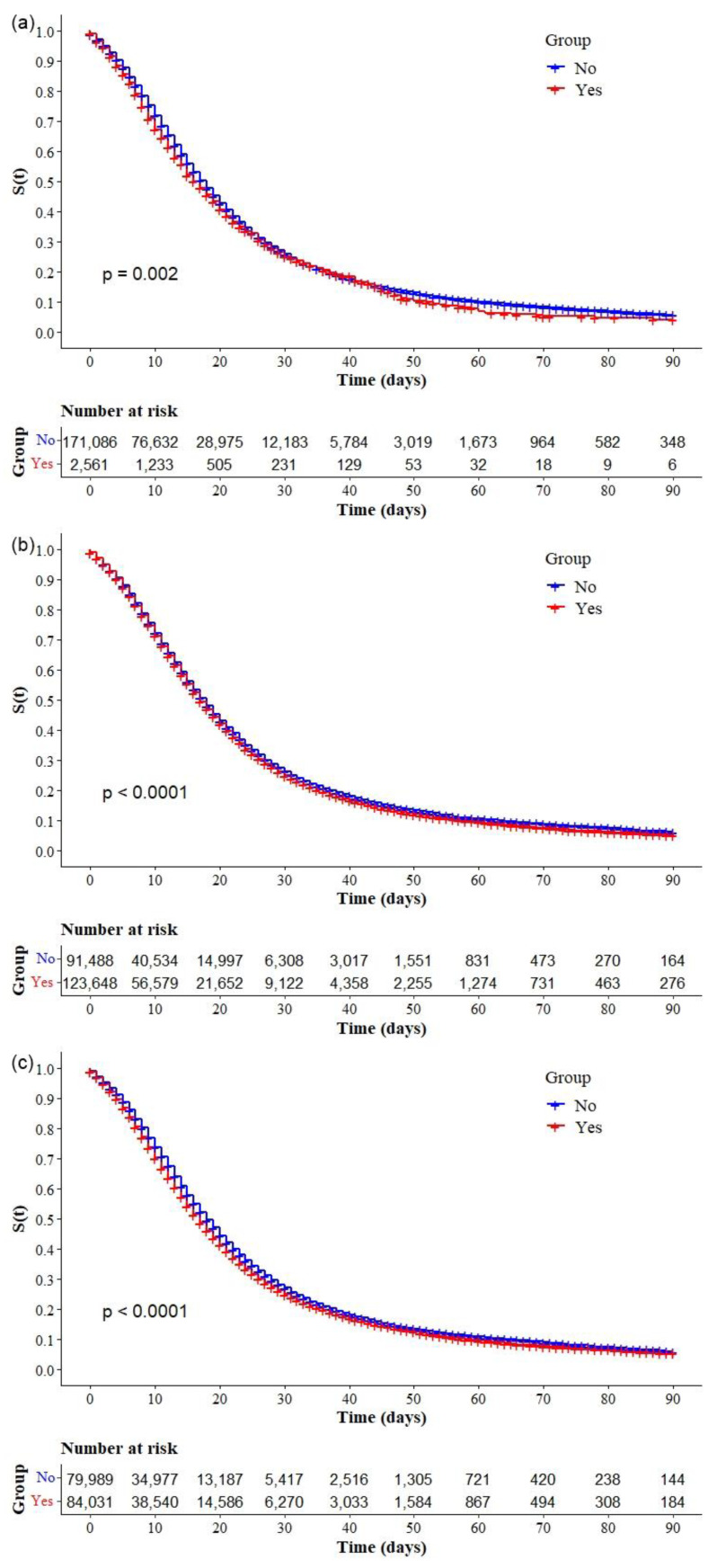
Kaplan–Meier survival curves for hospitalized COVID-19 adult patients in Brazil. Among all hospitalizations, the survival rate was stratified by the following comorbidities: (**a**) chronic hematological disease; (**b**) other comorbidity; and (**c**) multimorbidity.

**Figure 8:**
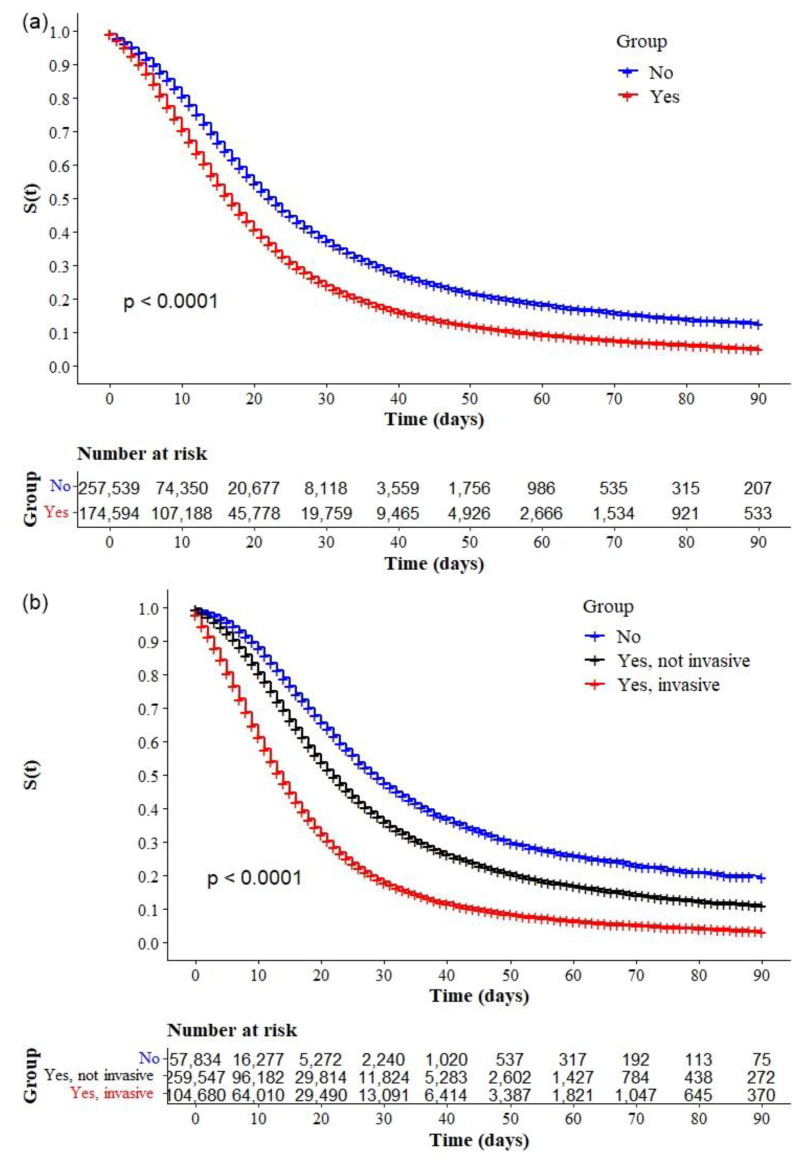
Kaplan–Meier survival curves for hospitalized COVID-19 adult patients in Brazil. Among all the hospitalized patients with COVID-19, the survival rate was stratified by (**a**) ICU admission and (**b**) ventilatory support (invasive or noninvasive).

The Kaplan–Meier survival curves showed lower survival in older, black, and mixed-race male individuals with low education levels (Figure 2a–d, respectively). Comparative analysis of the curves also showed that survival was lower in the north and northeast regions of Brazil (Figure 3). Survival function was also lower in subjects with heart disease, diabetes mellitus (Figure 4a,b), chronic kidney disease, chronic neurological disease, chronic lung disease (Figure 5a–c), immunodepression, chronic liver disease (Figure 6b,c), chronic hematological disease, and with other morbidities and multiple comorbidities (Figure 7a–c). The Kaplan–Meier bivariate analysis, in turn, showed that survival was shorter in individuals without obesity and asthma (Figure 4c and Figure 6a, respectively). Finally, lower survival was found in individuals admitted to ICU whether on noninvasive or invasive ventilatory support (Figure 8a,b).

Table S3 summarizes the bivariate analysis of the factors associated with in-hospital mortality from COVID-19 in Brazil. The results showed an association with all variables analyzed in the study (*p*-value < 0.05).

In the adjusted Cox regression analysis, we found an increase in mortality with increasing age. The risk of mortality was greater in age groups 40–49 years (aHR: 1.13; 95.0% CI: 1.02–1.25), 50–59 years (aHR: 1.20; 95.0% CI: 1.09–1.33), 60–69 years (aHR: 1.43; 95.0% CI: 1.30–1.57), and 70 years or more (aHR: 2.00; 95.0% CI: 1.82–2.20) when compared with individuals aged 18–29 years. We also found that the risk of mortality was higher in black (aHR: 1.06; 95.0% CI: 1.03–1.10) or mixed race (aHR: 1.07; 95.0% CI: 1.02–1.12) individuals; and adults with chronic liver disease (aHR: 1.22; 95.0% CI: 1.12–1.23), diabetes mellitus (aHR: 1.08; 95.0% CI: 1.06–1.10), chronic neurological disease (aHR: 1.18; 95.0% CI: 1.13–1.24), pneumopathy (aHR: 1.06; 95.0% CI: 1.01–1.11), immunodepression (aHR: 1.11; 95.0% CI: 1.06–1.18), chronic kidney disease (aHR: 1.14; 95.0% CI: 1.09–1.19), and obesity (aHR: 1.04; 95.0% CI: 1.00–1.07); and individuals using any ventilary support, noninvasive (aHR: 1.32; 95.0% CI: 1.26–1.39) or invasive (aHR: 2.20; 95.0% CI: 2.08–2.32). In contrast, the risk was lower in women (aHR: 0.94; 95.0% CI: 0.93–0.97), people with higher education (aHR = 0.64; 95.0% CI: 0.60–0.68), and people with asthma (aHR: 0.90; 95.0% CI: 0.84–0.95). When compared with the reference category (South region), we also observed that the risk was higher in all other regions, with greater magnitude in the North region (aHR: 1.37; 95.0% CI: 1.29–1.46) (Table 3).

**Figure 9:**
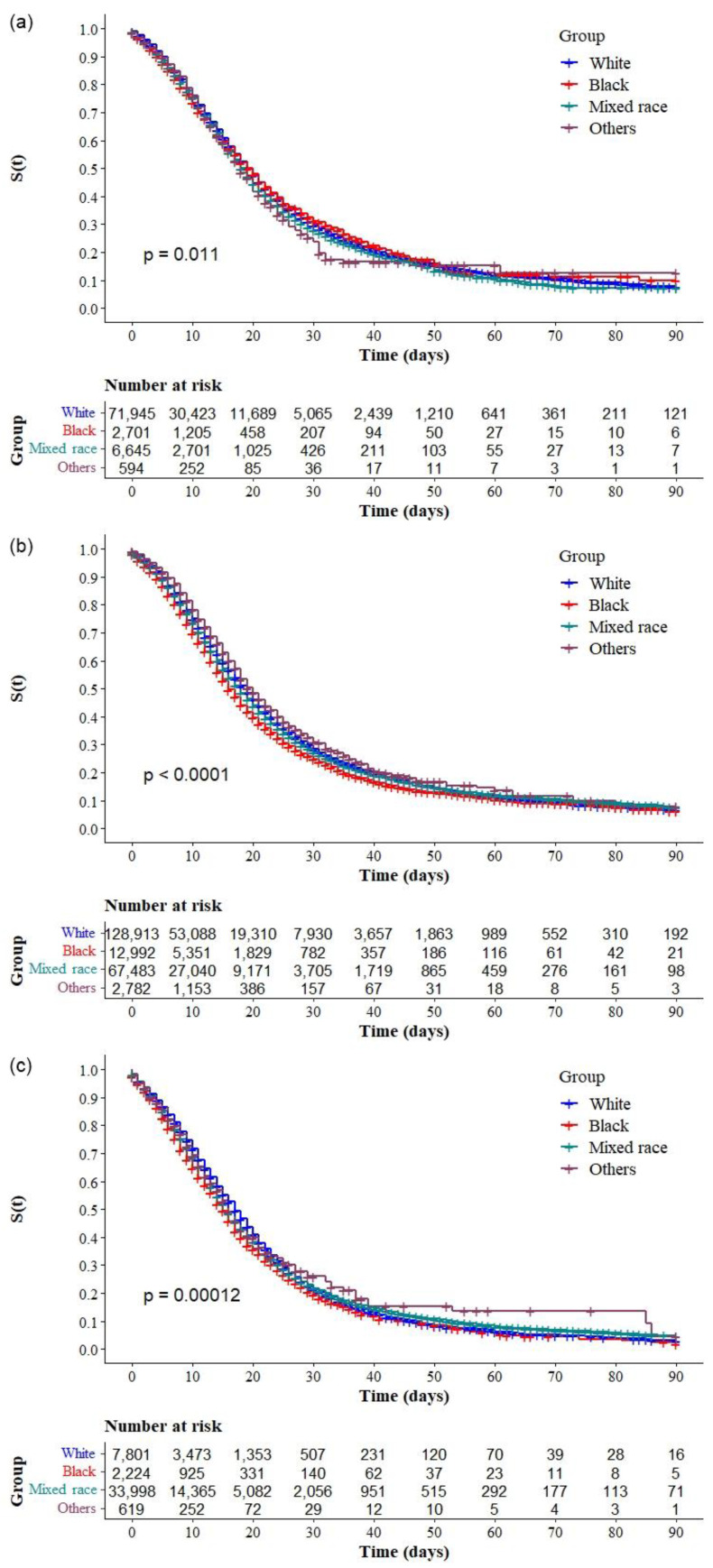
Kaplan–Meier survival curves for hospitalized COVID-19 adult patients in Brazil stratified by race and region: (**a**) South, (**b**) Southeast, and (**c**) Northeast.

**Figure 10:**
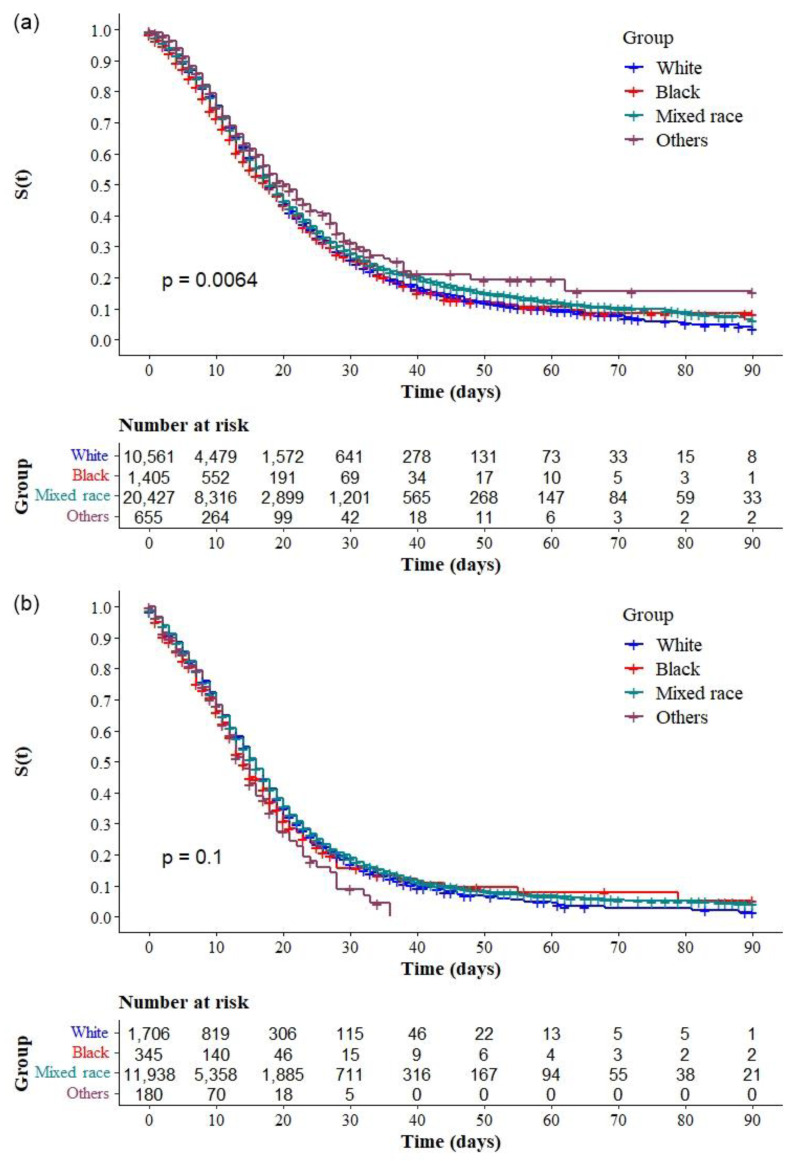
Kaplan–Meier survival curves for hospitalized COVID-19 adult patients in Brazil stratified by race and region: (**a**) Central-West, and (**b**) North.

Additional analyses comparing Kaplan-Meier curves according to race/ between each region were performed to understand the magnitude of the association between this variable and the survival of individuals with COVID-19 within the regions of Brazil. There were significant differences in the survival of patients with COVID-19 according to race/ in the South (Figure 9a), Southeast (Figure 9b), Northeast (Figure 9c), and Central-west (Figure 10a) regions, but none in the North (Figure 10b). Analysis of the log-rank test comparing pairs showed a statistically significant difference in the survival of hospitalized patients with COVID-19 between mixed-race and whites (*p* = 0.003) and between mixed-race and other races (*p* = 0.047) in the South: survival was lower in individuals of mixed race. Significant differences were found between blacks and whites (*p* < 0.001), blacks and mixed races (*p* < 0.001), and blacks and other races (*p* < 0.001), in addition to the differences between whites and mixed races (*p* < 0.001), whites and other races (*p* < 0.001), and mixed races and other races (*p* < 0.001) in the Southeast: survival was lower in black and mixed-race individuals. In the Central-West, there were differences between whites and other races (*p* = 0.024), blacks and mixed races (*p* = 0.012), and blacks and other races (*p* = 0.005): survival was lower in black individuals when compared with persons of mixed races or other races. Finally, in the Northeast, there was a significant difference between blacks and other races (*p* = 0.037), but no difference between whites and blacks or whites and mixed races. In the northeast, there was no significant difference in survival according to race.

We also assessed the influence of each sign or symptom of COVID-19 by performing bivariate (Figures S1–S4 & Table S3) and multiple regression (Table S4) analyses. The adjusted analysis showed that while mortality was higher in subjects with dyspnea, oxygen saturation <95%, respiratory distress, and abdominal pain, it was lower in subjects who reported fever, cough, diarrhea, fatigue, and changes in smell and taste (Table S4).

## 4. Discussion

This study analyzed data from 470,000 adults hospitalized with COVID-19 in Brazil. We found an in-hospital case fatality rate of 37.5%. Multiple regression analysis showed that older age, male sex, black and mixed race, and low level of education were associated with higher mortality risk. Chronic liver disease, diabetes mellitus, chronic neurological disease, pneumopathy, immunodepression, chronic kidney disease, and obesity increased the risk of mortality, while asthma was negatively associated with the outcome. As expected, individuals on invasive and noninvasive ventilatory support had a higher mortality risk. Finally, we found that dyspnea, oxygen saturation <95%, the presence of respiratory distress, and abdominal pain were risk factors for in-hospital mortality in patients.

Our results showed that older age and male sex were associated with in-hospital mortality, which is consistent with previous studies conducted in developed and developing countries such as Brazil [11,12,13,19,24,25]. Mortality risk increases with age due to progressively reduced innate and adaptive immunity, increased response efficiency to general infections, chronic low-grade inflammation, and higher prevalence of comorbidities (e.g., cardiovascular disease and diabetes) [26,27]. The higher mortality in men compared with women may be due to a still limited clinical evidence indicating that circulating levels of angiotensin-converting enzyme 2 (ACE2), one of the major receptors used by SARS-CoV-2 to enter cells and a major marker associated with organ failure and disease severity, is higher in men than in women [28,29]. Differences in inflammatory responses to other viral infections between sexes, the associations of inflammatory and immunologic statuses with comorbidities (e.g., cardiovascular disease and obesity), and the protection by genes expressed on the X chromosome and sex hormones that play a key role in immunity and adaptive roles in infection may also explain the difference in COVID-19 mortality found between sexes [28,29].

Moreover, it was found that black and mixed-race individuals, as well as those with low education levels, had higher mortality rates when compared with white individuals and those with high levels of education. These results corroborate previous studies conducted in Brazil [1,10,30]. There is still no solid evidence that race is associated with a higher risk of COVID-19. In Brazil, race is used as a proxy for the socioeconomic status of individuals; black and mixed-race people are more vulnerable when compared with white people, and that increases the likelihood of being infected with SARS-CoV-2. They also have limited access to health services (e.g., health insurance) and are more likely to have some comorbidities (e.g., cardiovascular diseases and diabetes) [10,11]. Moreover, black and mixed-race individuals may be admitted to hospitals with worse socioeconomic conditions and lower quality of care (e.g., access to ICU) when compared with white individuals, which increases the likelihood of mortality in these racial groups [1,31]. Other studies have investigated biological differences between race subgroups to explain the greater vulnerability of the black and mixed-race population for serious events resulting from COVID-19. We also found a positive gradient between increased mortality risk and level of education. Similar to results pertaining to race, a low level of education was associated with greater vulnerability to infection, limited access to health services, and a higher prevalence of morbidities, such as cardiovascular and respiratory diseases, which increases the risk of infection and disease severity [10,14,30].

We found that the mortality risk was higher in all regions than in the South Region, with the highest rates being observed in the north and northeast regions. This result is similar to those obtained in previous studies conducted in the country [1,19]. This result may reflect the socioeconomic and health care access differences among the regions of Brazil. The north and northeast are the least developed socioeconomically, having less access to health services and a lower number of hospital beds and ICU for critically ill patients, besides having a large deficit of health professionals, which can compromise the quality of care. Moreover, these regions have a higher proportion of black and mixed race individuals, which is a predictor variable of mortality and, therefore, a mediator of the association found, as these individuals have a higher burden of comorbidities and are more vulnerable to SARS-CoV-2 infection [1,19,32]. It is noteworthy that in January 2021, the emergence of the P.1 (gamma) variant became evident in Manaus, capital of the state of Amazonas (AM), a much more transmissible and aggressive variant that quickly spread throughout the states of the northern region, increasing the number of hospitalizations. The fact that the system of public hospitals linked to the Brazilian Unified Health System (SUS) is primarily responsible for meeting the health care needs of moderate to severe cases of COVID-19 may be another factor that explains the results found. In early 2021, the high demand for health care and the limitations of the system caused an unprecedented collapse in health services in several municipalities of Brazil, including Manaus, with the lack of oxygen and other essential supplies for the functioning of the ICUs, resulting in clinical worsening and the deaths of several patients [1,32,33].

Interestingly, the log-rank test for differences between race categories showed differences between mixed races and whites in the south; mixed races and whites and blacks and mixed races in the southeast; and blacks and mixed races in the central-west but no difference between whites and blacks and whites and mixed races in the northeast and north regions. This result indicates the impact of race on the survival of individuals with COVID-19 in the least developed regions of the country and that the difference in survival between regions is possibly due to the socioeconomic differences of the regions and accessibility of health resources.

Consistent with other studies, we found a higher mortality risk in individuals with some comorbidities [10,19,27] even after controlling for age in the regression model. Comorbidities such as cardiovascular disease, diabetes mellitus, chronic liver disease, chronic respiratory disease, chronic kidney disease, chronic neurological disease, and obesity have been reported by the Centers for Disease Control and Prevention and WHO and in meta-analyses as risk factors for severe COVID-19 and mortality, the latter only being found in some studies [16,17,27]. Particularly, metabolic diseases such as diabetes and obesity may increase the risk of clinical worsening and death from COVID-19 due to their suppressive action on innate and humoral immune responses, and they are associated with higher rates of ICU admission, use of invasive mechanical ventilation, and hospitalization [17,34]. Some authors observed that diabetic patients often have higher expression of ACE2, a receptor associated with the internalization of SARS-CoV-2, resulting from the use of oral hypoglycemic agents and greater secretion of cytokines, resulting in inflammation [17]. Moreover, studies show that these individuals are susceptible to severe chronic kidney disease marked by intense inflammatory response, thrombosis, and multiple organ failure due to a proinflammatory state, functional defects in innate and acquired immunity, and the frequent presence of comorbidities associated with COVID-19 (e.g., cardiovascular disease and diabetes) [16]. Although studies have shown the effect of chronic liver disease on mortality in hospitalized patients with COVID-19 due to immune response deficiency and inflammation, mortality may also occur due to the aggravation of SARS-CoV-2 in individuals with this comorbidity [35].

Our study has some limitations. First, the data do not include many important explanatory variables for the analysis of patient prognosis, especially clinical details, the medications and procedures used to treat the individuals, in-hospital complications, and the variants of the infected individuals. Second, the comorbidities were mostly self-reported, thus having low sensitivity and specificity, and they might be underestimated. Some variables such as the vaccination status of hospitalized patients were only added to the database after October 2021 and could not be analyzed as an independent variable for patient survival. Additionally, variables such as the patient variant are not available in public banks in Brazil. Third, many variables had missing data, which may have compromised the hazard ratios found in the analyses and may have omitted important risk factors for mortality. Fourth, underlying causes of death were not included in the database; thus, we used all-cause death as the outcome in mortality, not mortality from COVID-19. Fifth, cases and deaths of COVID-19 and SARS in Brazil are potentially underreported, especially in some regions of the country, hindering the inclusion of many cases in our analysis [36]. Sixth, the comorbidities reported in the database are not specific; for example, immunodepression may include people with human immunodeficiency virus (HIV) and cancer, which made the stratified analysis of these variables impossible. Finally, this study included only hospitalized patients, that is, those who are most prone to death, and therefore did not cover deaths that occurred outside of hospitals. As a result, the findings do not reflect the overall case fatality rate of COVID-19 individuals in Brazil, potentially underestimating or omitting important risk factors.

However, the study also has strengths, which include the use of a large population of hospitalized adults with COVID-19 in the cohort (~470,000). This increased the power of analysis by including data from public and private hospitals in Brazil. Despite the growing body of research on risk factors for COVID-19 mortality, few studies have assessed the predictors at the national level. This study also considered some confounding variables (e.g., race and level of education) that are important for the adjustment of the Cox regression model that were omitted in several studies. This analysis showed the influence of socioeconomic disparities on the COVID-19 mortality rate in Brazil. It also allowed for analyzing the contribution of each sign or symptom of COVID-19 to mortality risk, which has been little reported in the literature.

## 5. Conclusions

In conclusion, the analysis of a large cohort of adults hospitalized with COVID-19 in Brazil in 2021 showed that older age, with a linear increase with increasing age group; male sex; black and mixed race; low level of education; multiple comorbidities; and the use of any ventilatory support increased the risk of mortality. For signs and symptoms, dyspnea, oxygen saturation <95%, respiratory distress, and abdominal pain were also predictors of in-hospital mortality in patients. The results showed no significant differences in mortality between races in the less-developed northeast and north, but there were in the more developed regions (southeast, south and central-west), indicating that the difference in survival between regions is possibly due to the socioeconomic differences and accessibility of health resources.

These results contribute to public health policies in Brazil, suggesting the importance of promoting strategies to confront the pandemic, including improving non-pharmacological interventions, full universal vaccination coverage, and in-hospital care, focusing on subgroups and regions with higher vulnerability to the development of severe forms of COVID-19. Moreover, the surveillance and information systems must be improved, focusing on detailing some variables and including others, as well as implementing a system integrated with other systems such as the mortality system to allow a more robust analysis of the risk factors for mortality in Brazil. These interventions and future studies are critical for addressing the pandemic, especially given the emergence of new variants of greater transmissibility, more severe disease, significant reduction in neutralization by antibodies generated during previous infection or vaccination, reduced effectiveness of treatments or vaccines, or diagnostic detection failure, such as the variants B.1.1.7 (alpha), B.1.351 (beta) P.1. (gamma), B.1.617.1 (kappa), and B.1.617.2 (delta) [7].

## Figures and Tables

**Table 1:** Sociodemographic characteristics of hospitalized COVID-19 positive cases, Brazil, 2021.

Variables	
Age (years), median (IQR)—(n = 468,266) *	53 (41–75)
Age group (years), n (%)—(n = 468,266) *	
18–29	17,741 (3.8)
30–39	53,527 (11.4)
40–49	84,678 (18.1)
50–59	105,905 (22.6)
60–69	95,361 (20.4)
≥70	111,014 (23.7)
Sex, n (%)—(n = 468,184) *	
Male	261,227 (55.8)
Female	206,957 (44.2)
Race, n (%)—(n = 385,914) *	
White	220,926 (57.2)
Mixed race	140,491 (36.4)
Black	19,667 (5.1)
Others (Asian or Indigenous)	4830 (1.3)
Education, n (%) (n = 166,220) *	
Illiterate	7660 (4.6)
Elementary School (ES-1)	41,077 (24.7)
Elementary School (ES-2)	32,379 (19.5)
High School	58,675 (35.3)
Higher Education	26,429 (15.9)
Region country, n (%) (n = 468,226) *	
South	93,676 (20.0)
Southeast	255,258 (54.5)
Central-West	43,856 (9.4)
Northeast	59,981 (12.8)
North	15,455 (3.3)

* Numbers in parentheses (*n*) indicate the total number of valid responses for a given variable.

**Table 2:** Clinical characteristics of hospitalized COVID-19 positive cases, Brazil, 2021.

Variables	
*Comorbidities*, n (%)	
Cardiopathy (231,155) *	150,495 (65.1)
Diabetes mellitus (n = 213,403) *	103,011 (48.3)
Obesity (n = 189,113) *	51,189 (27.1)
Chronic kidney disease (n = 176,998) *	13,581 (7.7)
Chronic neurological disease (n = 176,881) *	13,046 (7.4)
Lung disease (n = 177,147) *	12,480 (7.0)
Asthma (n = 176,482) *	11,465 (6.5)
Immunodepression (n = 175,239) *	9129 (5.2)
Chronic liver disease (n = 173,330) *	3284 (1.9)
Chronic hematological disease (n = 173,647) *	2561 (1.5)
Other (n = 215,136) *	123,648 (57.5)
Multimorbidity, n (%) (n = 164,020) *	84,031 (51.2)
*Health assistance*, n (%)	
UTI admission, (n = 432,133) *	
No	257,539 (59.6)
Yes	174,594 (40.4)
Ventilatory support, (n = 422,061) *	
No	47,834 (13.7)
Yes, not invasive	259,547 (61.5)
Yes, invasive	104,680 (24.8)
*Signs or symptoms*, n (%)	
Fever (*n* = 389,388) *	258,970 (66.5)
Cough (*n* = 407,117) *	321,678 (79.0)
Sore throat (326,378) *	77,772 (23.8)
Dyspnea (*n* = 418,016) *	348,344 (83.3)
Respiratory distress (382,136) *	269,783 (70.6)
Oxygen < 95% saturation (*n* = 406,401) *	330,397 (81.3)
Diarrhea (*n* = 323,410) *	62,319 (19.3)
Vomit (*n* = 315,985) *	36,705 (11.6)
Abdominal pain (*n* = 309,346) *	26,393 (8.5)
Fatigue (*n* = 332,710) *	131,105 (39.4)
Alterations in smell (*n* = 313,009) *	45,969 (14.6)
Alterations in taste (*n* = 313,712) *	46,859 (14.9)

* Numbers in parentheses (*n*) indicate the total number of valid responses for a given variable. ICU = intensive care unit.

**Table 3:** Multiple regression analysis results for the risk factors for mortality in adults hospitalized with COVID-19 in Brazil, 2021.

Variables	aHR	95% CI	β	*p*-Value *
Age group (years)				
18–29	1.00			
30–39	1.07	0.96–1.19	0.053	0.206
40–49	1.13	1.02–1.25	0.040	0.019
50–59	1.20	1.09–1.33	0.049	<0.001
60–69	1.43	1.30–1.57	0.049	<0.001
≥70	2.00	1.82–2.20	0.049	<0.001
Sex				
Male	1.00			
Female	0.94	0.93–0.97	0.011	<0.001
Race				
White	1.00			
Mixed race	1.07	1.02–1.12	0.023	0.005
Black	1.06	1.03–1.10	0.015	<0.001
Others (Asian or Indigenous)	0.96	0.85–1.09	0.065	0.548
Education				
Illiterate	1.00			
Elementary School (ES-1)	0.94	0.84–0.98	0.024	0.009
Elementary School (ES-2)	0.83	0.79–0.88	0.025	<0.001
High School	0.77	0.73–0.80	0.025	<0.001
Higher Education	0.64	0.60–0.68	0.028	<0.001
Region country				
South	1.00			
Southeast	1.17	1.14–1.20	0.014	<0.001
Central-West	1.28	1.21–1.34	0.025	<0.001
Northeast	1.05	1.00–1.10	0.024	0.026
North	1.37	1.29–1.46	0.031	<0.001
*Comorbidities*				
Diabetes mellitus				
No	1.00			
Yes	1.08	1.06–1.10	0.011	<0.001
Obesity				
No	1.00			
Yes	1.04	1.00–1.07	0.015	0.018
Chronic kidney disease				
No	1.00			
Yes	1.14	1.09–1.19	0.022	<0.001
Chronic neurological disease				
No	1.00			
Yes	1.18	1.13–1.24	0.023	<0.001
Pneumopathy				
No	1.00			
Yes	1.06	1.01–1.11	0.024	0.012
Asthma				
No	1.00			
Yes	0.90	0.84–0.95	0.030	<0.001
Immunodepression				
No	1.00			
Yes	1.11	1.06–1.18	0.027	<0.001
Chronic liver disease				
No	1.00			
Yes	1.22	1.12–1.33	0.043	<0.001
*Health assistance*				
Ventilatory support				
No	1.00			
Yes, not invasive	1.32	1.26–1.39	0.026	<0.001
Yes, invasive	2.20	2.08–2.32	0.028	<0.001

Number of valid answers included in the model: 132,049. β = Regression coefficient; aHR = Adjusted hazard ratio; 95% CI: 95% confidence interval; * Wald test.

## Data Availability

The data used in this study are public and can be accessed on the website: https://opendatasus.saude.gov.br/dataset/srag-2021-e-2022 (accessed on 10 August 2021).

## Supplementary Materials

The following are available online at https://www.mdpi.com/article/10.3390/ijerph192114074/s1, Figure S1. Kaplan–Meier survival curves for hospitalized COVID-19 adult patients in Brazil. Among all hospitalizations, survival rate was stratified by the following clinical symptoms: (a) fever, (b) cough, and (c) sore throat. Figure S2. Kaplan–Meier survival curves for hospitalized COVID-19 adult patients in Brazil. Among all hospitalizations, survival rate was stratified by the following clinical symptoms: (a) dyspnea, (b) respiratory distress and (c) Oxygen < 95% saturation. Figure S3. Kaplan-Meier survival curves for hospitalized COVID-19 adult patients in Brazil. Among all hospitalizations, survival rate was stratified by the following clinical symptoms: (a) diarrhea, (b) vomiting and (c) abdominal pain. Figure S4. Kaplan–Meier survival curves for hospitalized COVID-19 adult patients in Brazil. Among all hospitalizations, survival rate was stratified by the following clinical symptoms: (a) fatigue, (b) changes in smell and (c) taste changes. Table S1. Population characteristics disaggregated by subgroups of survivors and death in Brazil, 2021. Table S2. Median survival time and 95% confidence interval by subgroups, Brazil, 2021. Table S3. Bivariate analysis of risk factors for mortality in adults hospitalized with COVID-19 in Brazil, 2021. Table S4. Multiple regression analysis of the signs and symptoms associated with mortality in adults hospitalized with COVID-19 in Brazil, 2021.
