# Supplementary material for: Mortality Risk Factors for Coronavirus Infection in Hospitalized Adults in Brazil: A Retrospective Cohort Study
Source: Int J Environ Res Public Health. 2022 Oct 28;19(21):14074. doi: 10.3390/ijerph192114074 (PMC9654637; doi:10.3390/ijerph192114074)
Supplement: Supplementary file 1 [file ijerph-19-14074-s001.zip › Supplementary Figures.pdf]

Figure S1. Kaplan–Meier survival curves for hospitalized COVID-19 adult patients in Brazil. Among all hospitalizations, survival rate was stratified by the following clinical symptoms: (a) fever, (b) cough, and (c) sore throat

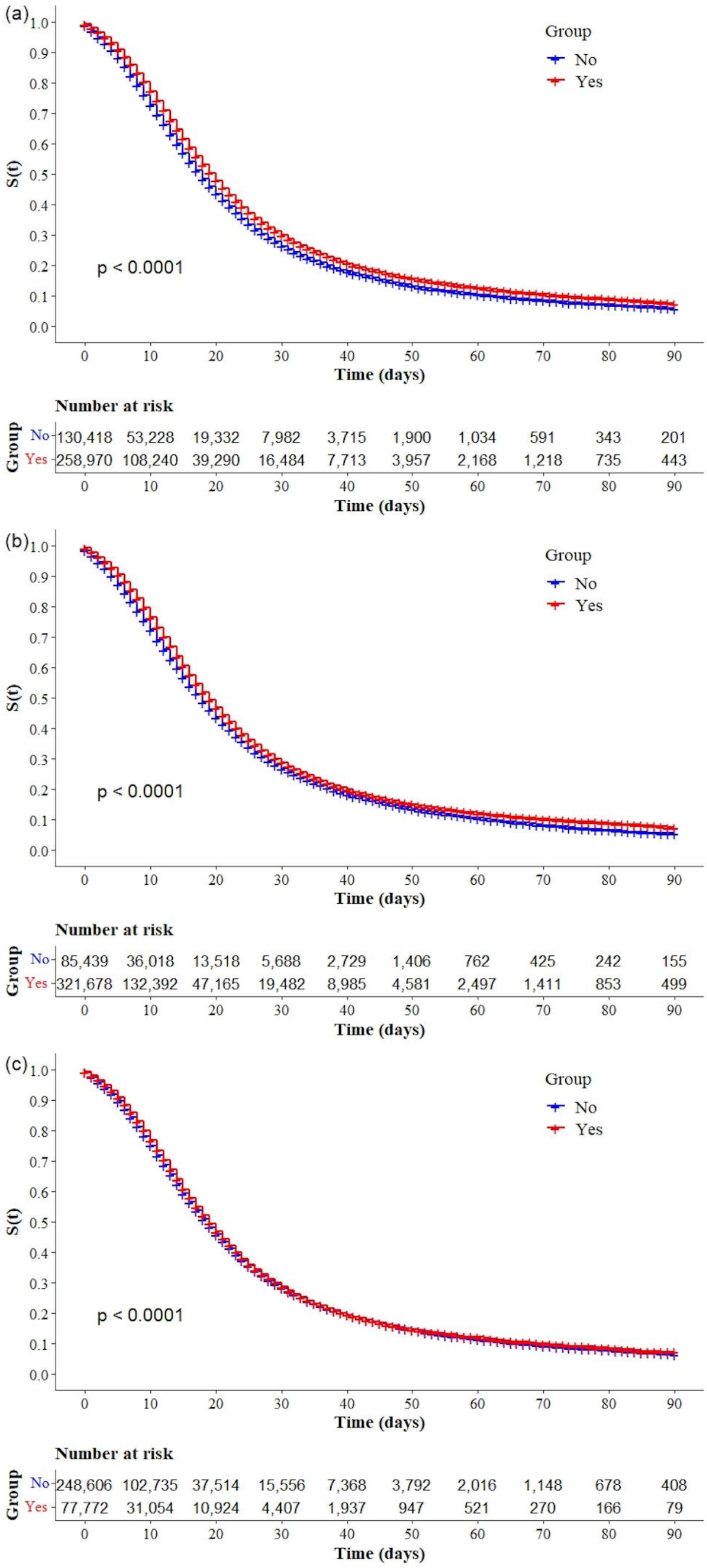
(a)
S(t)
Group
No
Yes
p < 0.0001
Time (days)
Number at risk
No 130,418 53,228 19,332 7,982 3,715 1,900 1,034 591 343 201
Yes 258,970 108,240 39,290 16,484 7,713 3,957 2,168 1,218 735 443
(b)
p < 0.0001
Number at risk
No 85,439 36,018 13,518 5,688 2,729 1,406 762 425 242 155
Yes 321,678 132,392 47,165 19,482 8,985 4,581 2,497 1,411 853 499
(c)
p < 0.0001
Number at risk
No 248,606 102,735 37,514 15,556 7,368 3,792 2,016 1,148 678 408
Yes 77,772 31,054 10,924 4,407 1,937 947 521 270 166 79

Figure S2. Kaplan–Meier survival curves for hospitalized COVID-19 adult patients in Brazil. Among all hospitalizations, survival rate was stratified by the following clinical symptoms: (a) dyspnea, (b) respiratory distress and (c) Oxygen < 95% saturation.

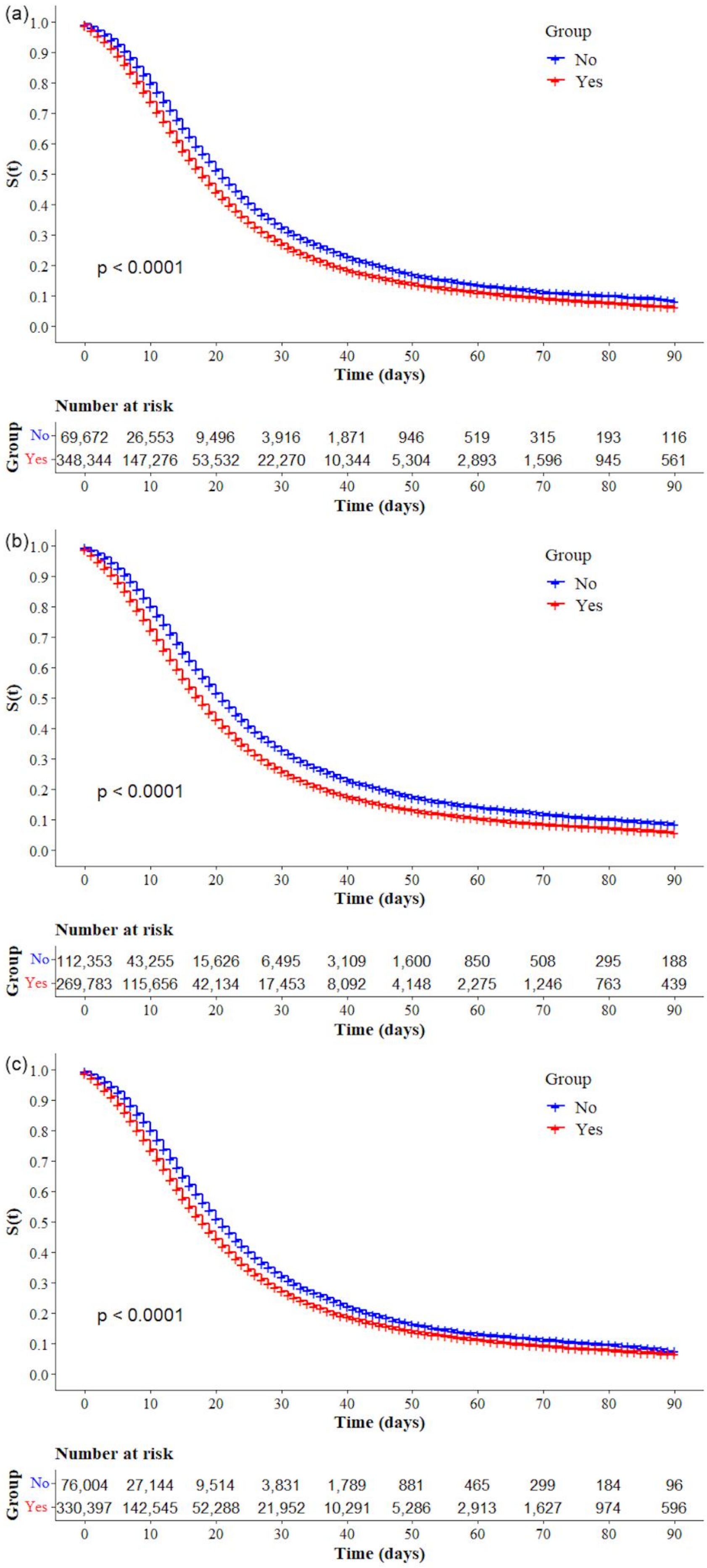
(a)
Group
No
Yes
S(t)
p < 0.0001
Time (days)
Number at risk
No 69,672 26,553 9,496 3,916 1,871 946 519 315 193 116
Yes 348,344 147,276 53,532 22,270 10,344 5,304 2,893 1,596 945 561
(b)
Group
No
Yes
S(t)
p < 0.0001
Time (days)
Number at risk
No 112,353 43,255 15,626 6,495 3,109 1,600 850 508 295 188
Yes 269,783 115,656 42,134 17,453 8,092 4,148 2,275 1,246 763 439
(c)
Group
No
Yes
S(t)
p < 0.0001
Time (days)
Number at risk
No 76,004 27,144 9,514 3,831 1,789 881 465 299 184 96
Yes 330,397 142,545 52,288 21,952 10,291 5,286 2,913 1,627 974 596

Figure S3. Kaplan–Meier survival curves for hospitalized COVID-19 adult patients in Brazil. Among all hospitalizations, survival rate was stratified by the following clinical symptoms: (a) diarrhea, (b) vomiting and (c) abdominal pain.

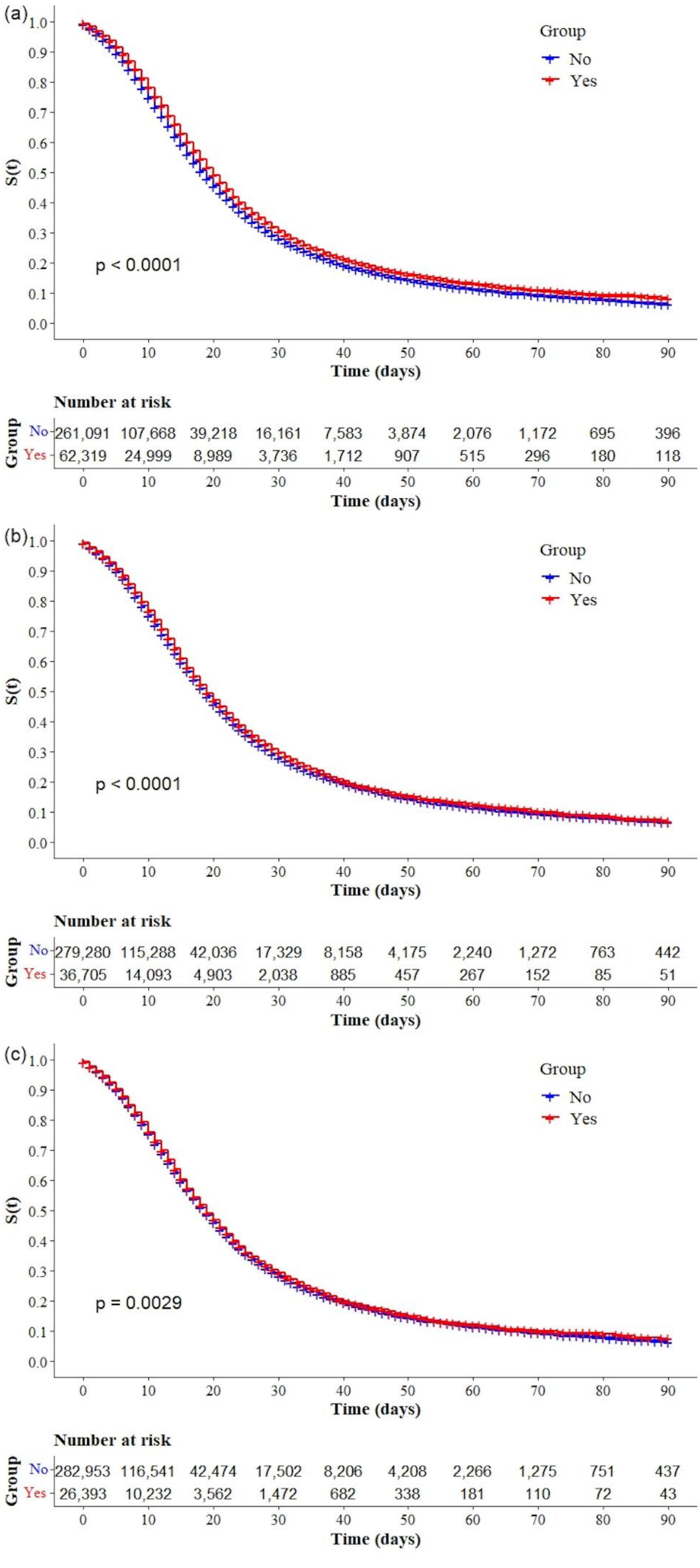
(a)
S(t)
1.0
0.9
0.8
0.7
0.6
0.5
0.4
0.3
0.2
0.1
0.0
Group
No
Yes
p < 0.0001
0 10 20 30 40 50 60 70 80 90
Time (days)
Number at risk
Group
No 261,091 107,668 39,218 16,161 7,583 3,874 2,076 1,172 695 396
Yes 62,319 24,999 8,989 3,736 1,712 907 515 296 180 118
0 10 20 30 40 50 60 70 80 90
Time (days)
(b)
S(t)
1.0
0.9
0.8
0.7
0.6
0.5
0.4
0.3
0.2
0.1
0.0
Group
No
Yes
p < 0.0001
0 10 20 30 40 50 60 70 80 90
Time (days)
Number at risk
Group
No 279,280 115,288 42,036 17,329 8,158 4,175 2,240 1,272 763 442
Yes 36,705 14,093 4,903 2,038 885 457 267 152 85 51
0 10 20 30 40 50 60 70 80 90
Time (days)
(c)
S(t)
1.0
0.9
0.8
0.7
0.6
0.5
0.4
0.3
0.2
0.1
0.0
Group
No
Yes
p = 0.0029
0 10 20 30 40 50 60 70 80 90
Time (days)
Number at risk
Group
No 282,953 116,541 42,474 17,502 8,206 4,208 2,266 1,275 751 437
Yes 26,393 10,232 3,562 1,472 682 338 181 110 72 43
0 10 20 30 40 50 60 70 80 90
Time (days)

Figure S4. Kaplan–Meier survival curves for hospitalized COVID-19 adult patients in Brazil. Among all hospitalizations, survival rate was stratified by the following clinical symptoms: (a) fatigue, (b) changes in smell and (c) taste changes.

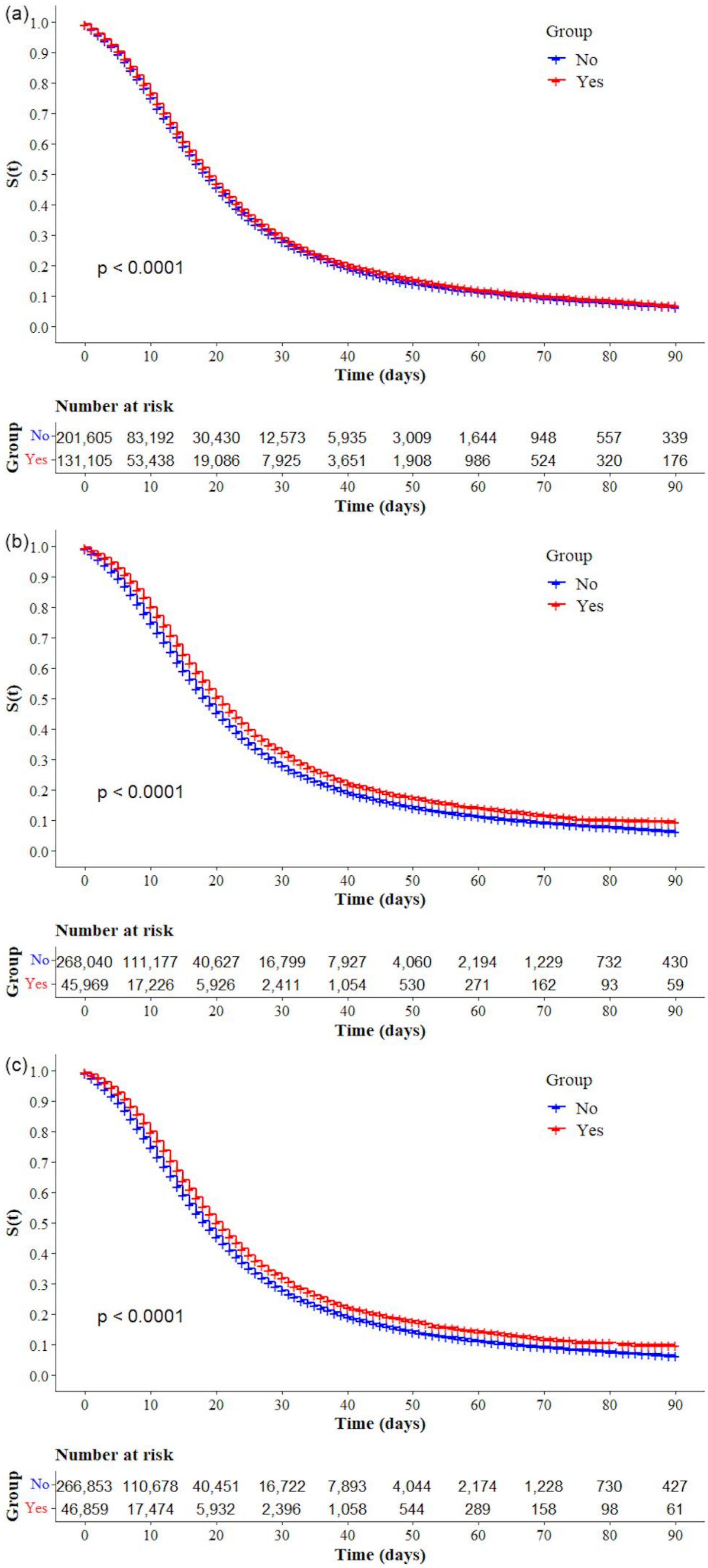
(a)
S(t)
Group
No
Yes
p < 0.0001
Time (days)
Number at risk
Group
No 201,605 83,192 30,430 12,573 5,935 3,009 1,644 948 557 339
Yes 131,105 53,438 19,086 7,925 3,651 1,908 986 524 320 176
Time (days)
(b)
S(t)
Group
No
Yes
p < 0.0001
Time (days)
Number at risk
Group
No 268,040 111,177 40,627 16,799 7,927 4,060 2,194 1,229 732 430
Yes 45,969 17,226 5,926 2,411 1,054 530 271 162 93 59
Time (days)
(c)
S(t)
Group
No
Yes
p < 0.0001
Time (days)
Number at risk
Group
No 266,853 110,678 40,451 16,722 7,893 4,044 2,174 1,228 730 427
Yes 46,859 17,474 5,932 2,396 1,058 544 289 158 98 61
Time (days)
