# Supplementary material for: Mortality Risk Factors for Coronavirus Infection in Hospitalized Adults in Brazil: A Retrospective Cohort Study
Source: Int J Environ Res Public Health. 2022 Oct 28;19(21):14074. doi: 10.3390/ijerph192114074 (PMC9654637; doi:10.3390/ijerph192114074)
Supplement: Supplementary file 1 [file ijerph-19-14074-s001.zip › Supplementary tables.pdf]

Tables

Table S1. Population Characteristics disaggregated by subgroups of survivors and death, Brazil, 2021

Variables	Cure	Death	p-value
Total	292,500	175,684	
Age (years), median (IQR)	53 (42-64)	65 (43-65)	<0.001*
Age group (years), n (%) -(n = 468,266)‡			
18-29	14,995 (5.1)	2,746 (1.6)	<0.001†
30-39	43,711 (14.9)	9,816 (5.6)	
40-49	64,414 (22.0)	20,264 (11.5)	
50-59	71,523 (24.5)	34,382 (19.6)	
60-69	52,526 (18.0)	42,835 (24.4)	
≥70	45,352 (15.5)	65,662 (37.4)	
Sex, n (%) - (n = 468,184) ‡			
Female	128,750 (44.0)	78,207 (44.5)	0.001†
Male	163,750 (56.0)	97,477 (55.5)	
Race, n (%)- (n = 385,914) ‡			
White	137,637 (58.5)	83,289 (55.3)	<0.001†
Mixed-race	83,487 (35.5)	57,004 (37.9)	
Black	11,104 (4.7)	8,563 (5.7)	
Others (Asian or Indigenous)	3,138 (1.3)	1,692 (1.1)	
Education, n (%) - (n = 166,220) ‡			
Illiterate	3,125 (3.1)	4,535 (6.8)	<0.001†
Elementary School (ES-1)	20,210 (20.3)	20,867 (31.3)	
Elementary School (ES-2)	18,454 (18.5)	13,925 (20.9)	
High School	39,188 (39.4)	19,487 (29.2)	
Higher Education	18,578 (18.7)	7,851 (11.8)	
Region country, n (%) - (n = 468,226) ‡			
South	58,994 (20.2)	34,682 (19.7)	<0.001†
Southeast	164,490 (56.2)	90,768 (51.7)	
Central-West	28,034 (9.6)	15,822 (9.0)	
Northeast	33,278 (11.4)	26,703 (15.2)	
North	7,725 (2.6)	7,730 (4.4)	
Signs or symptoms, n (%)			
Fever (n = 389,388) ‡	168,065 (68.1)	90,905 (63.7)	<0.001†

Cough (n =407,117) ‡	207,729 (80.6)	113,949 (76.3)	<0.001†
Sore throat (n = 326,378) ‡	51,290 (24.8)	26,482 (22.2)	<0.001†
Dyspnea (n = 418,016) ‡	210,377 (81.0)	137,967 (87.2)	<0.001†
Respiratory discomfort (n = 382,136) ‡	156,778 (66.4)	113,005 (77.4)	<0.001†
Oxygen< 95% saturation (n = 406,401) ‡	196,933 (78.1)	133,464 (86.5)	<0.001†
Diarrhea (n = 323,410) ‡	42,002 (20.5)	20,317 (17.2)	<0.001†
Vomit (n = 315,985) ‡	24,669 (12.3)	12,036 (10.4)	<0.001†
Abdominal pain (n = 309,346) ‡	17,503 (8.9)	8,890 (7.8)	<0.001†
Fatigue (n = 332,710) ‡	84,944 (40.2)	46,161 (38.0)	<0.001†
Olfactory changes (n = 313,009) ‡	32,947 (16.5)	13,022 (11.4)	<0.001†
Loss of taste (n = 313,712) ‡	33,576 (16.8)	13,283 (11.7)	<0.001†
Comorbidities, n (%)			<0.001†
Cardiopathy (n = 231,155)	78,982 (62.4)	71,513 (68.4)	<0.001†
Chronic hematological disease (n = 173,647) ‡	1,204 (1.2)	1,357 (1.8)	<0.001†
Chronic liver disease (n = 173,330) ‡	1,378 (1.4)	1,906 (2.5)	<0.001†
Asthma (n = 176,482) ‡	7,387 (7.5)	4,078 (5.2)	<0.001†
Diabetes mellitus (n = 213,403) ‡	52,288 (44.8)	50,723 (52.4)	<0.001†
Chronic neurological disease (n = 176,881) ‡	5,367 (5.5)	7,679 (9.7)	<0.001†
Pneumopathy (n = 177,147) ‡	5,241 (5.4)	7,239 (9.1)	<0.001†
Immunosuppression (n = 175,239) ‡	4,150 (4.3)	4,979 (6.4)	<0.001†
Chronic kidney disease (n = 176,998) ‡	5,099 (5.2)	8,482 (10.7)	<0.001†
Obesity (n = 189,113) ‡	28,248 (26.9)	22,941 (27.3)	<0.001†
Other morbidity (n = 215,136) ‡	66,505 (56.1)	57,143 (59.2)	<0.001†
Multimorbidity, n (%) (n = 164,020) ‡	44,515 (48.6)	39,516 (54.6)	<0.001†
Health assistance, n (%)			
ICU admission, n (%) - (n = 432,133) ‡			
No	203,802 (75.5)	53,737 (33.1)	<0.001†
Yes	66,063 (24.5)	108,531 (66.9)	
Ventilatory support, n (%) - (n = 422,061) ‡			
No	49,727 (18.8)	8,107 (5.1)	<0.001†
Yes, not invasive	193,163 (73.2)	66,384 (41.9)	
Yes, invasive	20,922 (7.9)	83,758 (52.9)	

ICU=Intensive Care Unit; *Mann-Whitney test; †Pearson's chi-squared test; ‡Numbers in parentheses (n) indicate the total number of valid responses for a given variable.

Table S2. Median survival time and 95% confidence interval by subgroups, Brazil, 2021

Variables	Median survival time (95%CI)	p-value*
Total	19.0 (18.9-19.1)	
Age group (years)		
18-29	28.0 (26.7-29.3)	<0.001
30-39	25.0 (24.5-25.5)	
40-49	24.0 (23.7-24.3)	
50-59	21.0 (20.8-21.2)	
60-69	18.0 (17.8-18.2)	
≥70	14.0 (13.9-14.1)	
Sex		
Female	19.0 (18.8-19.1)	<0.001
Male	19.0 (18.9-19.1)	
Race		
White	19.0 (18.9-19.1)	<0.001
Mixed-race	17.0 (16.7-17.3)	
Black	17.0 (16.9-17.1)	
Others (Asian or Indigenous)	19.0 (18.2-19.8)	
Education		
Illiterate	13.0 (12.6-13.4)	<0.001
Elementary School (ES-1)	15.0 (14.8-15.2)	
Elementary School (ES-2)	17.0 (16.7-17.3)	
High School	20.0 (19.7-20.3)	
Higher Education	22.0 (21.6-22.4)	
Region country		
South	19.0 (18.8-19.2)	<0.001
Southeast	19.0 (18.9-19.1)	
Central-West	19.0 (18.7-19.3)	
Northeast	17.0 (16.8-17.2)	
North	16.0 (15.7-16.3)	
Signs or symptoms		
Fever		
No	18.0 (17.9-18.1)	<0.001
Yes	20.0 (19.9-20.1)	

Cough		
No	18.0 (17.8-18.2)	<0.001
Yes	19.0 (18.9-19.1)	
Sore throat		
No	19.0 (18.9-19.1)	<0.001
Yes	19.0 (18.8-19.2)	
Dyspnea		
No	21.0 (20.7-21.3)	<0.001
Yes	18.0 (17.9-18.1)	
Respiratory discomfort		
No	21.0 (20.8-21.2)	<0.001
Yes	18.0 (17.9-18.1)	
Oxygen< 95% saturation		
No	21.0 (20.7-21.3)	<0.001
Yes	18.0 (17.9-18.1)	
Diarrhea		
No	19.0 (18.9-19.1)	<0.001
Yes	20.0 (19.8-20.2)	
Vomit		
No	19.0 (18.9-19.1)	<0.001
Yes	19.0 (18.7-19.3)	
Abdominal pain		
No	19.0 (18.9-19.1)	0.003
Yes	19.0 (18.6-19.4)	
Fatigue		
No	19.0 (18.9-19.2)	<0.001
Yes	19.0 (18.8-19.2)	
Olfactory changes		
No	19.0 (18.9-19.1)	<0.001
Yes	21.0 (20.7-21.3)	
Loss of taste		
No	19.0 (18.9-19.1)	<0.001
Yes	21.0 (20.7-21.3)	
Comorbidities		

Cardiopathy		
No	18.0 (17.8-18.2)	<0.001
Yes	17.0 (16.9-17.1)	
Chronic hematological disease		
No	18.0 (17.9-18.1)	<0.001
Yes	17.0 (16.0-18.0)	
Chronic liver disease		
No	18.0 (17.9-18.1)	<0.001
Yes	15.0 (14.2-15.8)	
Asthma		
No	17.0 (16.9-17.2)	<0.001
Yes	21.0 (20.4-21.6)	
Diabetes mellitus		
No	18.0 (17.9-18.2)	<0.001
Yes	16.0 (15.9-16.1)	
Chronic neurological disease		
No	18.0 (17.9-18.2)	<0.001
Yes	14.0 (13.6-14.4)	
Pneumopathy		
No	18.0 (17.9-18.1)	<0.001
Yes	15.0 (14.6-15.4)	
Immunosuppression		
No	18.0 (17.9-18.1)	<0.001
Yes	17.0 (16.5-17.5)	
Chronic kidney disease		
No	18.0 (17.9-18.1)	<0.001
Yes	14.0 (13.7-14.3)	
Obesity		
No	17.0 (16.9-17.2)	<0.001
Yes	18.0 (17.8-18.2)	
Other morbidity		
No	18.0 (17.8-18.2)	<0.001
Yes	17.0 (16.9-17.1)	
Multimorbidity		

No	18.0 (17.8-18.2)	<0.001
Yes	17.0 (16.8-17.2)	
Health service		
ICU admission		
No	23.0 (22.8-23.2)	<0.001
Yes	17.0 (16.9-17.2)	
Ventilatory support		
No	29.0 (28.3-29.7)	<0.001
Yes, not invasive	22.0 (21.8-22.2)	
Yes, invasive	14.0 (13.9-14.1)	

95%CI: 95% Confidence Interval; ICU: Intensive care unit; *Log-rank test.

Table S3. Bivariate analysis of risk factors for mortality in adults hospitalized with COVID-19 in Brazil, 2021

Variables	cHR	95%CI	p-value*
Age group (years)			
18-29	1.00		
30-39	1.11	1.07-1.16	<0.001
40-49	1.30	1.25-1.35	<0.001
50-59	1.56	1.50-1.62	<0.001
60-69	1.96	1.88-2.03	<0.001
≥ 70	2.83	2.72-2.94	<0.001
Sex			
Female	1.00		
Male	1.03	1.02-1.04	<0.001
Race			
White	1.00		
Mixed race	1.18	1.15-1.20	<0.001
Black	1.11	1.10-1.13	<0.001
Others (Asian or Indigenous)	0.98	0.93-1.02	0.311
Education			
Illiterate	1.00		
Elementary School (ES-1)	0.82	0.80-0.86	<0.001
Elementary School (ES-2)	0.69	0.66-0.71	<0.001
High School	0.55	0.53-0.57	<0.001
Higher Education	0.45	0.44-0.47	<0.001
Region country			
South	1.00		
Southeast	1.00	0.99-1.02	0.347
Central-West	0.99	0.98-1.01	0.567
Northeast	1.23	1.20-1.25	<0.001
North	1.33	1.30-1.36	<0.001
Comorbidities			
Cardiopathy			
No	1.00		
Yes	1.12	1.10-1.13	<0.001

Diabetes mellitus			
No	1.00		
Yes	1.16	1.15-1.18	<0.001
Obesity			
No	1.00		
Yes	0.93	0.91-0.94	<0.001
Chronic kidney disease			
No	1.00		
Yes	1.27	1.24-1.30	<0.001
Chronic neurological disease			
No	1.00		
Yes	1.30	1.27-1.33	<0.001
Pneumopathy			
No	1.00		
Yes	1.25	1.22-1.28	<0.001
Asthma			
No	1.00		
Yes	0.80	0.77-0.83	<0.001
Immunosuppression			
No	1.00		
Yes	1.07	1.04-1.10	<0.001
Chronic liver disease			
No	1.00		
Yes	1.20	1.15-1.35	<0.001
Chronic hematological disease			
No	1.00		
Yes	1.08	1.03-1.15	0.002
Others			
No	1.00		
Yes	1.04	1.02-1.05	<0.001
Multimorbidity			
No	1.00		
Yes	1.10	1.09-1.12	<0.001
Health			

ICU admission			
No	1.00		
Yes	1.50	1.49-1.52	<0.001
Ventilatory support			
No	1.00		
Yes, not invasive	1.53	1.49-1.56	<0.001
Yes, invasive	2.91	2.84-2.98	<0.001

cHR=Crude Hazard Ratio; 95%CI: 95% Confidence Interval; *Wald test.

Table S4. Multiple regression analysis of the signs and symptoms associated with à mortality in adults hospitalized with COVID-19 in Brazil, 2021

Signs and symptoms	aHR	95% CI	β	p-value*
Fever				
No	1.00			
Yes	0.88	0.87-0.90	0.123	<0.001
Cough				
No	1.00			
Yes	0.90	0.89-0.92	0.101	<0.001
Sore throat				
No	1.00			
Yes	1.00	0.98-1.01	0.003	0.649
Dyspnoea				
No	1.00			
Yes	1.12	1.10-1.14	0.113	<0.001
O_2<95% saturation				
No	1.00			
Yes	1.10	1.09-1.12	0.100	<0.001
respiratory distress				
No	1.00			
Yes	1.26	1.24-1.28	0.230	<0.001
Diarrhoea				
No	1.00			
Yes	0.94	0.92-0.96	0.063	<0.001
Vomit				
No	1.00			
Yes	0.99	0.97-1.02	0.007	0.545
Abdominal pain				
No	1.00			
Yes	1.03	1.00-1.06	0.032	0.017
Fatigue				
No	1.00			
Yes	0.93	0.92-0.94	0.071	<0.001
Anosmia				

No	1.00			
Yes	0.92	0.89-0.94	0.089	<0.001
Ageusia				
No	1.00			
Yes	0.92	0.89-0.95	0.086	<0.001

β= Regression coefficient; aHR=Adjusted Hazard Ratio; 95%CI: 95% Confidence Interval; *Wald test.
